# Human γδ T-Cell Control of Mucosal Immunity and Inflammation

**DOI:** 10.3389/fimmu.2018.00985

**Published:** 2018-05-07

**Authors:** Neil E. McCarthy, Matthias Eberl

**Affiliations:** ^1^Centre for Immunobiology, Bart’s and The London School of Medicine and Dentistry, The Blizard Institute, Queen Mary University of London, London, United Kingdom; ^2^Division of Infection and Immunity, School of Medicine, Systems Immunity Research Institute, Cardiff University, Cardiff, United Kingdom

**Keywords:** human, mucosal, gammadelta T-cells, Vdelta1, Vdelta2

## Abstract

Human γδ T-cells include some of the most common “antigen-specific” cell types in peripheral blood and are enriched yet further at mucosal barrier sites where microbial infection and tumors often originate. While the γδ T-cell compartment includes multiple subsets with highly flexible effector functions, human mucosal tissues are dominated by host stress-responsive Vδ1^+^ T-cells and microbe-responsive Vδ2^+^ T-cells. Widely recognized for their potent cytotoxicity, emerging data suggest that γδ T-cells also exert strong influences on downstream adaptive immunity to pathogens and tumors, in particular *via* activation of antigen-presenting cells and/or direct stimulation of other mucosal leukocytes. These unique functional attributes and lack of MHC restriction have prompted considerable interest in therapeutic targeting of γδ T-cells. Indeed, several drugs already in clinical use, including vedolizumab, infliximab, and azathioprine, likely owe their efficacy in part to modulation of γδ T-cell function. Recent clinical trials of Vδ2^+^ T-cell-selective treatments indicate a good safety profile in human patients, and efficacy is set to increase as more potent/targeted drugs continue to be developed. Key advances will include identifying methods of directing γδ T-cell recruitment to specific tissues to enhance host protection against invading pathogens, or alternatively, retaining these cells in the circulation to limit peripheral inflammation and/or improve responses to blood malignancies. Human γδ T-cell control of mucosal immunity is likely exerted *via* multiple mechanisms that induce diverse responses in other types of tissue-resident leukocytes. Understanding the microenvironmental signals that regulate these functions will be critical to the development of new γδ T-cell-based therapies.

## Introduction

The γδ T-cell compartment includes some of the most numerous “antigen-specific” cell types in peripheral blood and is enriched yet further in mucosal tissues including the lung and intestine (1, 2). In humans, γδ T-cells are typically divided into distinct subsets based on δ-chain usage, each being specialized to detect a different class of common antigen or host molecule generated by microbial infection, stress, and/or malignant transformation. Pathobionts frequently invade the body *via* epithelial barriers, which are also major sites of tumorigenesis, hence γδ T-cell function in mucosal tissues represents a critical component of host protection against a range of major diseases. While the ability of human γδ T-cells to lyse infected or transformed host cells has been well documented, less is known about their influence on downstream antimicrobial immunity and mucosal inflammation, which must be carefully regulated in order to prevent autoimmune pathology, tissue damage, and cancer. Indeed, a recent analysis of tumor transcriptome data identified γδ T-cell infiltration as the best prognostic marker of survival (1), indicating that γδ T-cell responses can significantly influence clinical outcomes in human patients, but the mucosal functions of these cells and their impact on barrier protection remain poorly understood. This mini-review focuses on the potential roles of γδ T-cells in human mucosal tissues, with an emphasis on their ability to influence conventional leukocyte responses at these sites. We consider that γδ T-cell detection of stress molecules and microbial signals can significantly alter adaptive immunity and inflammation at mucosal barrier sites, consistent with the increasing recognition that tissue-resident T-cells play essential roles in human immunity. Where useful context has been drawn from studies performed in animal models, the non-human origins of these data have been clearly indicated.

## γδ T-Cells Mediate Epithelial Barrier Protection

Epithelial cells are exposed to a variety of microbial and environmental signals that induce distinct patterns of cytokine and chemokine secretion, as well as rapid changes in cell surface expression of host stress molecules. Acting in concert, these factors can stimulate a range of leukocyte responses as complex as those imparted by myeloid antigen-presenting cells (3). Innate-like lymphocytes residing in the epithelial layer and underlying mucosa are key responders to these barrier stress signals, and γδ T-cells comprise a major component of this “unconventional” lymphocyte pool. It is well-established that epithelial signaling to γδ T-cells begins early, in the thymus, where these cells are imparted with greater gut-homing potential (integrin α4β7 expression) than conventional lymphocytes, and exhibit more efficient proliferation upon subsequent recruitment to the murine mucosa (4). Less clear is how far epithelial cells continue to shape γδ T-cell function upon their arrival in mucosal tissues, although an intimate functional relationship controlled by a variety of different signals seems increasingly likely (5). Indeed, the γδ T-cell repertoire in human intestine undergoes major changes with age and becomes oligoclonal in adults (6), suggesting strong local selection by site-specific signals that include host butyrophilin-like molecules (5, 7), dietary and microbial ligands for the aryl hydrocarbon receptor (8), and common pathogen products and stress antigens. Accordingly, studies in parabiotic mice have demonstrated that the frequency of γδ T-cell mixing between animals is low in the gut epithelium, whereas up to 50% cell exchange between animals can be observed in the lamina propria (9). These data suggest that Vδ1^+^ intraepithelial lymphocytes (γδ-IEL) may develop *in situ*, whereas lamina propria γδ T-cells depend both on recruitment from the peripheral blood and local proliferation in order to maintain the local pool. In mice, it is widely accepted that the majority of γδ T-cells are pre-programmed with cytokine potential and effector functions within the thymus (10). However, recent data suggest that γδ T-cell function outside the thymus is more plastic than originally thought (11), and the murine Vγ7^+^ subset appears to require Btnl1 expression by the gut epithelium to develop IFNγ-expressing capacity (5). In humans, the closely related proteins BTNL3 and BTNL8 may similarly cooperate to promote colonic expansion of the analogous Vγ4^+^ subset (5), although the functional impact of this mechanism remains unclear, and populations expressing alternative γ-chains also reside in this tissue. Nonetheless, human γδ T-cell function does not appear to be “hard-wired” in the thymus and remains receptive to site-specific cues that likely induce distinct functional profiles in different tissues and organs. Intriguingly, BTNL2 is primarily expressed in the small intestinal epithelium and appears to function as a negative regulator of T-cell activation (12), with mutations in this protein conferring increased risk of inflammatory bowel disease (IBD) (13). It is possible, therefore, that therapeutic strategies targeting BTNL molecules and/or γδ T-cell activation in the human gut may yield new treatment options for patients with IBD.

Consistent with a role for γδ-IEL in monitoring gut barrier function, recent data indicate that these cells are highly motile in the mouse intestine and actively scan the epithelium for signs of cellular stress, with pro-inflammatory cytokines and/or pathogen encounter significantly modulating this behavior (14, 15). Indeed, while γδ-IEL numbers appear largely unaffected in germ-free mice (16), epithelial cell contact with gut bacteria can induce γδ-IEL expression of antimicrobial peptides (17), confirming that exposure to the microbiota can significantly alter their function. It is likely that human gut γδ-IELs scan the epithelium for expression of MHC I-related genes MICA and MICB, which function as stress-inducible triggers for γδ T-cell cytotoxicity (18, 19). MICA/B expression has already been identified in carcinomas of the lung and colon, where these molecules are associated with enhanced tumor infiltration by cytotoxic γδ T-cells (20). Accordingly, γδ T-cells isolated from human lung tumors can selectively lyse autologous malignant cells *ex vivo* (21). Vδ1^+^ γδ T-cells also seem to be expanded in many transplant recipients, where they express gut-homing receptors and are strongly activated by intestinal tumor cells but not healthy epithelial cell lines (22).

MICA/B is recognized with high affinity by the natural killer (NK) cell receptor NKG2D (23), which is expressed by human γδ-IELs under the control of IL-15 (24). This cytokine appears to play an important role in steady-state maintenance of the murine γδ-IEL compartment (25), and thymic expression of IL-15 is required to modulate histone acetylation of the Vγ5 gene segment, which is preferentially used by mouse gut γδ-IELs (26). Consistent with these data is the observation that epithelial supply of IL-15 cytokine plays a crucial role in γδ T-cell control of mucosal inflammation in murine colitis (27). Similarly, human intestinal Vδ1^+^ T-cells are significantly expanded in both celiac disease and IBD (28, 29), which are characterized by high mucosal levels of the tissue damage-associated cytokine IL-15 (30–32). Intriguingly, patients with celiac disease exhibit upregulated activity of cytotoxic lymphocytes (24), but a subset of NKG2A^+^ γδ T-cells can reportedly decrease IFNγ expression by cocultured gut αβ T-cells (33). Similarly, transfer of γδ-IELs into mice that lack these cells can protect against chemical colitis by decreasing host lymphocyte expression of pro-inflammatory cytokines and modulating epithelial production of IL-15 (27). These data strongly suggest that γδ-IELs help maintain the integrity of the epithelium by altering the local activity of other gut leukocyte subsets, and that IL-15 may alert these cells to tissue stress, including the need to remove infected/malignant epithelial cells from the barrier. Consequently, when intestinal γδ T-cells are deleted in murine models, the gut epithelium displays uncontrolled IFNγ expression, chronic inflammation, and impaired barrier regeneration (34).

Vδ1^+^ T-cell influences on other leukocyte populations have previously been reported in various settings of relevance to mucosal barrier protection. For example, maturation of CD1c^+^ myeloid dendritic cells (DC) can be induced by direct contact with CD1c-restricted Vδ1^+^ T-cells *in vitro* (35), suggesting that similar interactions may also occur at mucosal sites *in vivo*. The resultant mature, CD1c^+^ DC are endocytic, can efficiently present novel protein antigens, and are more potent stimulators of naïve T-cell proliferation than DC activated with cytokines alone. Intriguingly, these characteristics can also be observed in human lung DC isolated from patients with atopic asthma and may represent genuine features of mucosal inflammatory disorders (36). Vδ1^+^ T-cell-induced maturation of CD1^+^ myeloid DC does not rely on foreign antigen and is chiefly mediated by TNFα (35), which also triggers rapid activation of γδ T-cells (37–39), and likely enables timely immune responses to a barrier breach. Indeed, full DC maturation has long been known to require interaction with T-cells (40), but αβ T-cell clones with fine antigen specificity are rare in the periphery. Tissue-resident γδ T-cells may therefore accelerate DC maturation in the mucosa by relying on non-polymorphic molecules to mediate this interaction (41). Indeed, increased CD1 expression by APC has already been reported to enhance T-cell stimulation in a murine model (42). It is also important to note that this model does not preclude a role for the microbiota, since microbial antigen can enhance APC presentation of self-antigens to CD1-restricted T-cells (43). Indeed, CD1^+^ DC can also present pollen-derived lipid antigens to Vδ1^+^ T-cells derived from the blood of allergic donors (44), suggesting that similar interactions can also occur in the human lung in allergic asthma. While it is unclear to what extent laboratory mice can accurately model asthma pathology (45), previous studies have observed a major influence of lung γδ T-cells on allergen-induced airway hyperreactivity and excess production of IgE (46, 47), which are cardinal features of the human disease. Intriguingly, these effects were again associated with a shift in cytokine production by pulmonary αβ T-cells, further suggesting that γδ T-cells can exert complex effects *via* their influence on other mucosal leukocyte populations. Indeed, gut-tropic γδ T-cells can promote Th1/Th17 differentiation of CD4^+^ T-cells *in vivo* to exacerbate colitis in murine models (48, 49), and a Vδ2^+^ subset expressing the PD1 isoform Δ42 promotes gut inflammation in humanized mice *via* putative effects on DC (50). Together, these data suggest that γδ T-cells may exert similarly potent influences on adaptive immunity and inflammation in human mucosal tissues.

## γδ T-Cells Stimulate Complex Mucosal Leukocyte Responses

Often referred to as rare cells, recent estimates suggest that the phosphoantigen-responsive Vδ2^+^ population in fact accounts for ~1 in 40 memory T-cells in healthy adults and may represent the single largest recall response in the human body (2, 51). Indeed, Vδ2^+^ T-cells are capable of expanding yet further to dominate the blood lymphocyte pool in a wide range of infections (52, 53), which has led to extensive study of these cells in the circulation as well as the common misconception that they are restricted to the blood. However, several reports have now identified that the majority of blood Vδ2^+^ T-cells express homing receptors for epithelial barrier sites including the skin (CLA) and intestine (integrin α4β7 and CCR9) (50, 54, 55). This tissue-tropic phenotype is consistent with the role of Vδ2^+^ T-cells in host protection against pathogens that colonize epithelial barriers and produce the metabolite (*E*)-4-hydroxy-3-methyl-but-2-enyl pyrophosphate (HMB-PP) (56). Indeed, while circulating Vδ2^+^ T-cells are well situated to detect host cell accumulation of phosphoantigen in blood malignancies (57), the majority of non-hematological cancers are epithelial in origin, such that Vδ2^+^ T-cell recruitment to these sites is likely to enhance tumor surveillance as well as antimicrobial immunity. Vδ2^+^ T-cells express high levels of α4β7 in humans (22, 55, 58), are rapidly recruited to mucosal tissues in higher primates *in vivo* (59, 60), and mediate effective host protection against bacteria in humanized mice (61). Recent work in a macaque model also demonstrated that injection of HMB-PP or related compounds stimulates Vδ2^+^ T-cell expansion in the blood and accumulation of a CD27^+^ IFNγ-producing subset in the lungs (59). Intriguingly, lung accumulation of Vδ2^+^ T-cells persisted for several months and was associated with a corresponding increase in CD4^+^ and CD8^+^ conventional T-cell numbers, suggesting that activation of mucosal Vδ2^+^ T-cells exerts multiple downstream effects on other leukocyte compartments. Indeed, Vδ2^+^ T-cell activation *in vivo* has since been shown to enhance conventional Th1 responses in the lung and promote mucosal release of growth factors that confer protection against a range of different pathogens (including *Listeria monocytogenes, Mycobacterium tuberculosis*, and *Yersinia pestis*) (60, 62, 63). Since HMB-PP injection into macaques promotes Vδ2^+^ T-cell expansion and recruitment to the intestinal mucosa as well as the lung (59, 60), it is likely that these cells exert similar effects on αβ T-cell responses and antimicrobial immunity in the primate gut. Consistent with this concept, human gut tissue contains Vδ2^+^ T-cells that express the tissue-resident memory T-cell marker CD103, exhibit distinct patterns of cytokine production, and modify IFNγ expression by autologous gut CD4^+^ T-cells (55, 64). In mice, T-cell entry into the epithelium combined with local IL-15 and TGF-β signaling is required for the formation of long-lived memory cells that express CD103 (65). Whether or not CD103^+^ Vδ2^+^ T-cells in human tissues represent a long-lived population with distinct roles in mucosal immunity is currently unclear.

We have previously demonstrated that activation of Vδ2^+^ T-cells in human intestine modulates cytokine production by colonic αβ T-cells in the same piece of gut tissue (55), indicating that these cells are present in sufficient numbers to exert potent effects on downstream mucosal immunity. Moreover, like Vδ1^+^ T-cells the Vδ2^+^ population can promote generation of mature DC *via* a TNF-dependent mechanism (66, 67), illustrating a marked ability of these “innate-like” cells to trigger adaptive immune responses. In the case of the Vδ2^+^ subset, this process also confers potent APC capacity on the γδ T-cells, likely allowing rapid amplification of immune responses at sites of barrier breach and microbial invasion. Early work in this area demonstrated that microbial activation induced human Vδ2^+^ T-cells to process and present antigens as efficiently as DC, as well as provide co-stimulatory signals that stimulated naïve αβ T-cell proliferation and differentiation (68, 69). It is now widely recognized that blood Vδ2^+^ T-cells can display remarkably flexible APC functions, while the nature of the αβ T-cell responses they induce in tissues is likely directed by the stimuli encountered at specific anatomical sites. Indeed, “Vδ2-APC” function appears to be optimally induced by microenvironmental signals known to be highly enriched in the human gut, namely microbe-derived HMB-PP (70), pro-inflammatory mediator TNFα (71), and epithelial cytokine IL-15 (32). It is perhaps unsurprising then that human intestinal Vδ2-APC are efficient inducers of the barrier defense mediator IL-22 (72), whereas conventional myeloid APC in this tissue are instead specialized to induce “pro-symbiotic” IL-17 responses (73). Intriguingly, therapeutic antibody-mediated disruption of Th17 biology led to increased mucosal inflammation in patients with Crohn’s disease during randomized controlled trials (74, 75), suggesting that Vδ2^+^ T-cell-directed immunotherapies might prove to be an effective method of enhancing barrier protection without impacting on mucosal levels of IL-17. Indeed, recruitment of circulating Vδ2^+^ T-cells to inflamed skin lesions has already been identified in patients with psoriasis (54), and this population can also infiltrate the peritoneal cavity in patients with bacterial infections (39). In both cases, local Vδ2^+^ T-cell numbers and activation state were significantly correlated with therapeutic/patient outcomes, suggesting that these cells significantly impact on the clinical course of both inflammatory and infectious pathologies affecting multiple human tissues.

## Future Considerations for Therapeutic Targeting of Mucosal γδ T-Cells

**Figure 1:**
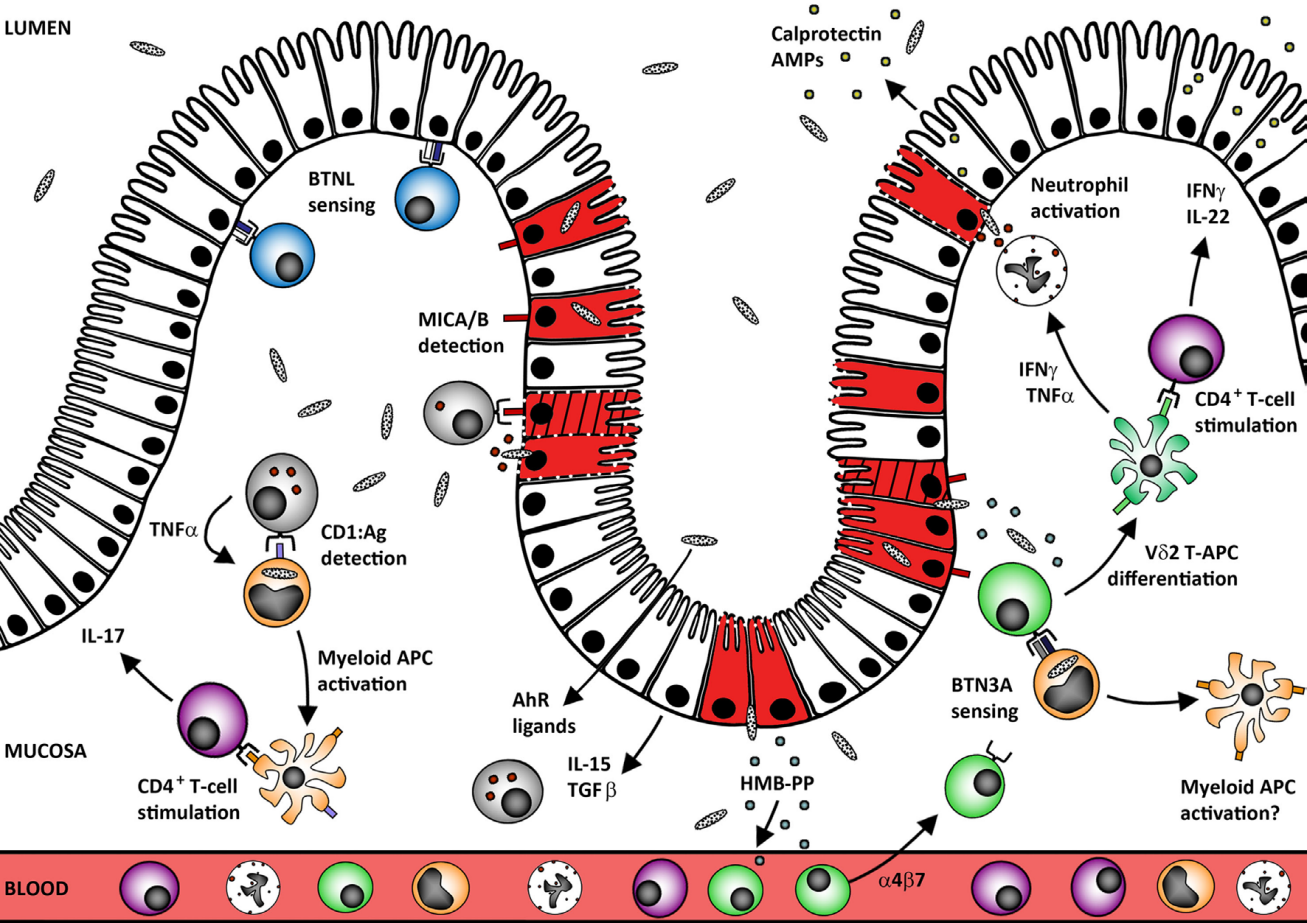
Human mucosal γδ T-cells protect the epithelial barrier against microbes and tumors. Tissue-resident γδ T-cells may develop *in situ* under the control of site-specific BTNL heterodimers that maintain these cells in a primed but inactivate state (blue Vδ1^+^ cells). Human mucosal barrier sites are also enriched in CD1^+^ myeloid APC (orange cells) that capture microbes and may undergo local TNF-induced maturation *via* self-antigen presentation to CD1-restricted γδ T-cells. The resultant mature APC can stimulate conventional αβ T-cell responses at the site of infection without the need to migrate through the draining lymphatics. Loss of BTNL signaling or upregulation of MICA/B expression by the infected/transformed/stressed epithelium (red/hatched/membrane-damaged cells) also triggers γδ T-cell cytotoxic responses that rapidly lyse the compromised cells (gray cells; both Vδ1^+^ and Vδ2^+^ subsets). Maintenance of these “epithelial surveillance” γδ T-cell populations is regulated by a complex variety of signals including local provision of AhR ligands, epithelial cytokine IL-15, and growth factor TGF-β. These factors likely also play critical roles in promoting tissue residence of recruited γδ T-cell populations. In the case of Vδ2^+^ T-cells (green cells), recruitment from the blood could be driven by (*E*)-4-hydroxy-3-methyl-but-2-enyl pyrophosphate (HMB-PP) translocation across the defective mucosal barrier. Accumulation of microbial HMB-PP in the mucosa can then trigger BTN3A-mediated activation of Vδ2^+^ T-cells in the presence of IL-15 to promote differentiation into potent APC (and perhaps also reciprocal activation of local myeloid cell populations). This process supports rapid local generation of presenting cells that can stimulate CD4^+^ T-cell expression of barrier protectant cytokines, including IFNγ and IL-22 (purple CD4^+^ T-cells). These mediators promote epithelial release of antimicrobial peptides (AMPs) including calprotectin and cooperate with TNFα to promote neutrophil activation and survival.

Human γδ T-cells display potent effector functions when exposed to microbial antigens and/or host molecules commonly encountered at barrier sites, but accumulating evidence suggests an additional ability to modulate downstream mucosal leukocyte responses (Figure 1). These features may be shared by multiple γδ T-cell subsets in human tissues, since even the little-studied Vδ3^+^ subset appears capable of complex patterns of cytokine expression and promoting DC maturation mediated by CD1/TNFα (76). Together, these data suggest that tissue-resident γδ T-cells play important roles in activating host immunity to microbes across multiple mucosal sites, not just the lung and intestine. Indeed, recent findings indicate that commensals residing in the ocular mucosa can induce γδ T-cell expression of IL-17 to drive neutrophil recruitment and protect the mouse eye against bacterial and fungal pathogens (77). Human γδ T-cells in mucosal tissues may be similarly specialized to detect local microbial and host stress molecules and respond not only with rapid effector functions, but also by relaying critical information to other mucosal leukocyte populations. Data from our own laboratories indicate that human Vδ2^+^ T-cells can significantly modify intestinal immune responses *via* direct antigen presentation *in vitro* (72), and influence the clinical outcome of microbial infections *in vivo* (39), hence these cells should be a high priority for the development of novel immunotherapies. Indeed, a recent transcriptome analysis of 585 human colorectal cancer samples revealed that tumor infiltration by IFNγ-producing Vδ2^+^ T-cells in particular was associated with higher probability of 5-year disease-free survival (78). Given that γδ T-cells also exhibit potent activity in non-malignant settings, it seems likely that therapies targeting these cells could prove effective in a range of different pathologies. Multiple drugs already in widespread clinical use likely owe their therapeutic efficacy in part to modulation of γδ T-cell function, including the anti-α4β7 antibody vedolizumab (79–81), anti-TNF agents including infliximab (38, 82–85), and immunosuppressant drug azathioprine (86). The aminobisphosphonate drug zoledronate has also been shown to promote Vδ2^+^ T-cell activation *in vivo* by inhibiting the farnesyl pyrophosphate synthase enzyme to allow host cell accumulation of isopentenylpyrophosphate (87). Work is ongoing to identify optimal strategies for zoledronate adjunctive therapy, which has so far displayed variable patient benefit in clinical trials (88, 89). However, the continuing development of aminobisphosphonate pro-drugs and γδ-selective nanobody agonists/antagonists will soon yield more potent therapies for a variety of major human disorders (90–92). Given that γδ T-cell biology is closely associated with epithelial barriers, a key consideration for future treatment strategies will be ensuring the effective targeting of γδ T-cells to tissues of interest (55, 58, 93, 94). Limiting γδ T-cell egress from the blood may prove beneficial when treating blood malignancies and mucosal inflammatory disorders such as IBD (e.g., with anti-integrin antibodies), whereas enhancing cell migration to barrier sites will likely be key to enhancing protection against mucosal infection and epithelial tumors. The extent to which therapeutic outcomes are influenced by the mechanisms that promote γδ T-cell tissue residency will also need to be explored.

## Author Contributions

NM and ME drafted the manuscript, revised the content, and approved the final version.

## Conflict of Interest Statement

The authors declare that the research was conducted in the absence of any commercial or financial relationships that could be construed as a potential conflict of interest.
